# Longitudinal Associations Between Inflammation and Multi‐Dimensional Fatigue up to 2 Years After Colorectal Cancer Diagnosis

**DOI:** 10.1002/ijc.70613

**Published:** 2026-06-19

**Authors:** Anouk E. C. Bruijnzeels, Floortje Mols, Katrijn Van Deun, Dounya Schoormans

**Affiliations:** ^1^ Department of Medical and Clinical Psychology, CoRPS – Center of Research on Psychological Disorders and Somatic Diseases, Tilburg School of Social and Behavioral Sciences Tilburg University Tilburg the Netherlands; ^2^ Department of Research & Development Netherlands Comprehensive Cancer Organisation (IKNL) Utrecht the Netherlands; ^3^ Department of Methodology and Statistics, Tilburg School of Social and Behavioral Sciences Tilburg University Tilburg the Netherlands

**Keywords:** biomarker, cancer‐related fatigue, colorectal cancer, PROCORE, survivorship

## Abstract

Cancer‐related fatigue (CRF) is a prevalent symptom among colorectal cancer (CRC) survivors. While inflammation is a proposed underlying mechanism, longitudinal evidence including pre‐treatment assessments remains scarce. Within the population‐based PROCORE study, newly diagnosed CRC patients provided blood samples and completed questionnaires at diagnosis (*n* = 411; 60.6% male; age = 67.0 years), 12‐ (*n* = 304), and 24‐month follow‐up (*n* = 252). Eleven inflammatory biomarkers (CRP, IFN‐γ, IL‐1α, IL‐1β, IL‐6, IL‐8, IL‐10, IL‐17A, IL‐22, sTNFRI, and sTNFRII) were assayed; CRF was measured with the Multidimensional Fatigue Inventory. Hybrid linear mixed models disentangled between‐ and within‐subject associations, controlling for sociodemographic (e.g., age), clinical (e.g., cancer treatment), and lifestyle covariates (e.g., BMI), sleep quality, and pain. A normative age‐ and sex matched sample (*n* = 204; 52.5% male; age = 64.3 years) was included for comparison. Soluble TNF receptors (sTNFRI/II) were most robustly and positively associated with nearly all fatigue dimensions. CRP was positively associated with mental and physical fatigue; IL‐8 positively associated with multiple domains including reduced motivation; and IFN‐γ positively associated with general fatigue and reduced activity. Lower IL‐1α was associated with more mental fatigue. Between‐subject effects mirrored overall results; within‐subject effects were more selective. Associations were most consistently observed for mental fatigue. In controls, less associations were significant; CRP was the most robust marker and positively associated with general fatigue, reduced activity, and reduced motivation. CRC survivors exhibited a broader, mostly TNF‐α driven inflammatory signature of fatigue than controls. Findings highlight inflammation as a potential target underlying CRF, informing survivorship care strategies.

## What's New?

Cancer‐related fatigue (CRF) is a common and debilitating symptom among colorectal cancer (CRC) patients, but its underlying biological causes remain unclear, with inflammation emerging as a leading suspect. Here, researchers analyzed inflammatory biomarkers in blood samples and evaluated responses in fatigue assessments from CRC patients at and up to two years after diagnosis, using statistical models to distinguish trait‐like vulnerability from short‐term symptom fluctuations. TNF‐α‐related signaling showed the strongest and most consistent association with fatigue, particularly mental fatigue. These relationships were less pronounced in controls. The findings identify inflammation as a potential driver of CRF and a target for more personalized care.

## Graphical abstract

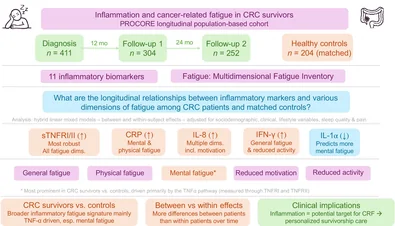

**Abbreviations**
*β*
: standardized regression coefficient
BMI: body mass index
CI: confidence interval
CRC: colorectal cancer
CRF: cancer‐related fatigue
CRP: C‐reactive protein
CT: chemotherapy
DeFaCT: Deep Learning and Understanding of Fatigue in Colorectal Cancer Patients: A Computational Bio‐Medical Psychology Approach
df: degrees of freedom
EORTC QLQ‐C30: European Organisation for Research and Treatment of Cancer Quality of Life Questionnaire C30
FDR: false discovery rate
FU: follow‐up
hsCRP: high sensitivity C‐reactive protein
IFN‐γ: interferon gamma
IL‐10: interleukin 10
IL‐17A: interleukin 17A
IL‐1α: interleukin 1 alpha
IL‐1β: interleukin 1 beta
IL‐22: interleukin 22
IL‐6: interleukin 6
IL‐8: interleukin 8
LISS: Longitudinal Internet studies for the Social Sciences
LMM: linear mixed‐effects model
M: mean
MFI: Multidimensional Fatigue Inventory
NCR: Netherlands Cancer Registry
PRO: patient‐reported outcome
PROFILES: Patient‐Reported Outcomes Following Initial Treatment and Long‐term Evaluation of Survivorship
PSQI: Pittsburgh Sleep Quality Index
RT: radiotherapy
SD: standard deviation
SE: standard error
sTNFRI: soluble tumor necrosis factor receptor 1
sTNFRII: soluble tumor necrosis factor receptor 2
TNF‐α: tumor necrosis factor alpha

## 1 Introduction

The number of colorectal cancer (CRC) survivors in the Netherlands has significantly increased over the past years due to advances in cancer treatments, better understanding of individual risk factors for CRC such as comorbidity and lifestyle behaviors (i.e., physical activity, smoking), improved screening, and better pre‐ and postoperative care [1]. While these developments have led to higher survival rates, CRC survivors continue to experience a range of long‐term side effects, which include cancer‐related fatigue (CRF), pain, and sleep problems. These symptoms frequently occur together in so‐called symptom clusters, with CRF as the most central component [2].

CRF is a debilitating symptom and differs from “normal” fatigue in the way that it persists, even with enough sleep and rest, and is unproportional in relation to recently performed activity [3]. It manifests as a subjective feeling of physical, emotional, and/or mental tiredness or exhaustion that is associated with cancer or its treatment, and interferes with usual functioning [4]. Importantly, CRF is mostly considered a multidimensional construct, typically encompassing physical, mental, and cognitive fatigue in definitions across studies [5]. CRF affects a large proportion of CRC patients, with prevalences up to 70% [5]. Despite CRF's pervasive impact, the underlying mechanisms remain poorly understood, making it difficult to develop targeted interventions to alleviate this symptom.

The etiology of CRF appears to be multifactorial, with biological, psychological, and clinical factors proposed as contributing elements [3, 6]. One biological mechanism that has received increasing attention is inflammation. Altered immune and inflammatory responses have been proposed as potential drivers of CRF [6], with elevated levels of pro‐inflammatory biomarkers such as C‐reactive protein (CRP), interleukin (IL)‐6, and tumor necrosis factor alpha (TNF‐α) linked to CRF across various cancer populations [3, 7, 8]. A study among patients with advanced cancer further suggests that different CRF dimensions may be associated with distinct inflammatory profiles, with physical fatigue in particular associated with increased CRP and IL‐6 levels [9].

To date, most studies on inflammation and CRF have been cross‐sectional, which limits conclusions about temporal directionality. Querido et al. [8] provided the first longitudinal evidence linking inflammation to CRF in CRC survivors in the EnCoRe study. In 257 patients with stage I‐III disease, higher hsCRP remained independently associated with greater CRF up to 12 months posttreatment, mainly for total subjective fatigue and reduced motivation subscales [8].

Building on the work of the EnCoRe study by Querido et al. [8], the current study extends the field by (i) including a pretreatment baseline to capture immune status before treatment interventions, allowing differentiation between pre‐existing inflammation and treatment‐related immune alterations, (ii) assessing a wider biomarker panel to provide a more comprehensive representation of inflammatory pathways, including cytokine signaling and acute‐phase responses potentially involved in CRF, (iii) employing a multidimensional view of CRF to capture distinct dimensions of fatigue that may relate to different underlying biological mechanisms, such as inflammation‐driven sickness behavior [9, 10], and (iv) incorporating an age‐ and sex‐matched cancer‐free control group. The latter is particularly important because inflammation is not unique to cancer but also shaped by aging, obesity, and comorbidities, enabling us to disentangle CRC‐specific effects from general health‐related processes [11, 12]. We therefore examine longitudinal relationships between inflammatory markers and various dimensions of fatigue from diagnosis up to 2 years later among CRC patients and matched controls, thereby informing the development of personalized, interdisciplinary treatment strategies to improve survivors' quality of life. By additionally distinguishing between effects of overall (between‐subject) differences in inflammation and within‐subject changes over time, the analyses of the current study enable us to disentangle whether CRF is primarily driven by stable interindividual differences (between‐subject associations) or by dynamic fluctuations in inflammatory activity (within‐subject associations).

## 2 Method

### 2.1 Setting, Participants, and Study Design

This study is part of the DeFaCT (Deep Learning and Understanding of Fatigue in Colorectal Cancer Patients: A Computational Bio‐Medical Psychology Approach) project and uses data from the PROCORE study, a longitudinal population‐based study evaluating the impact of CRC (treatment) on patient‐reported outcomes (PROs), including CRF [13, 14]. PROCORE data were collected via the PROFILES (Patient‐Reported Outcomes Following Initial Treatment and Long‐term Evaluation of Survivorship) registry, linked to the Netherlands Cancer Registry (NCR) [13, 14]. Details on data collection are described elsewhere [13, 14]. In brief, patients with a primary CRC diagnosis between January 2016 and January 2019 were recruited from four Dutch hospitals (Elisabeth‐TweeSteden Hospital, Catharina Hospital, Elkerliek Hospital, and Máxima Medical Center). Eligibility criteria further included fluency in Dutch and sufficient cognitive capacity to participate. Exclusion criteria encompassed previous cancer diagnoses (except basal cell carcinoma) and pre‐existing cancer treatment [13, 14]. Patients were recruited shortly after diagnosis and before treatment initiation with an average of 22.1 days after diagnosis and a maximum of 99 days after diagnosis.

### 2.2 Data Collection and Blood Processing

**FIGURE 1:**
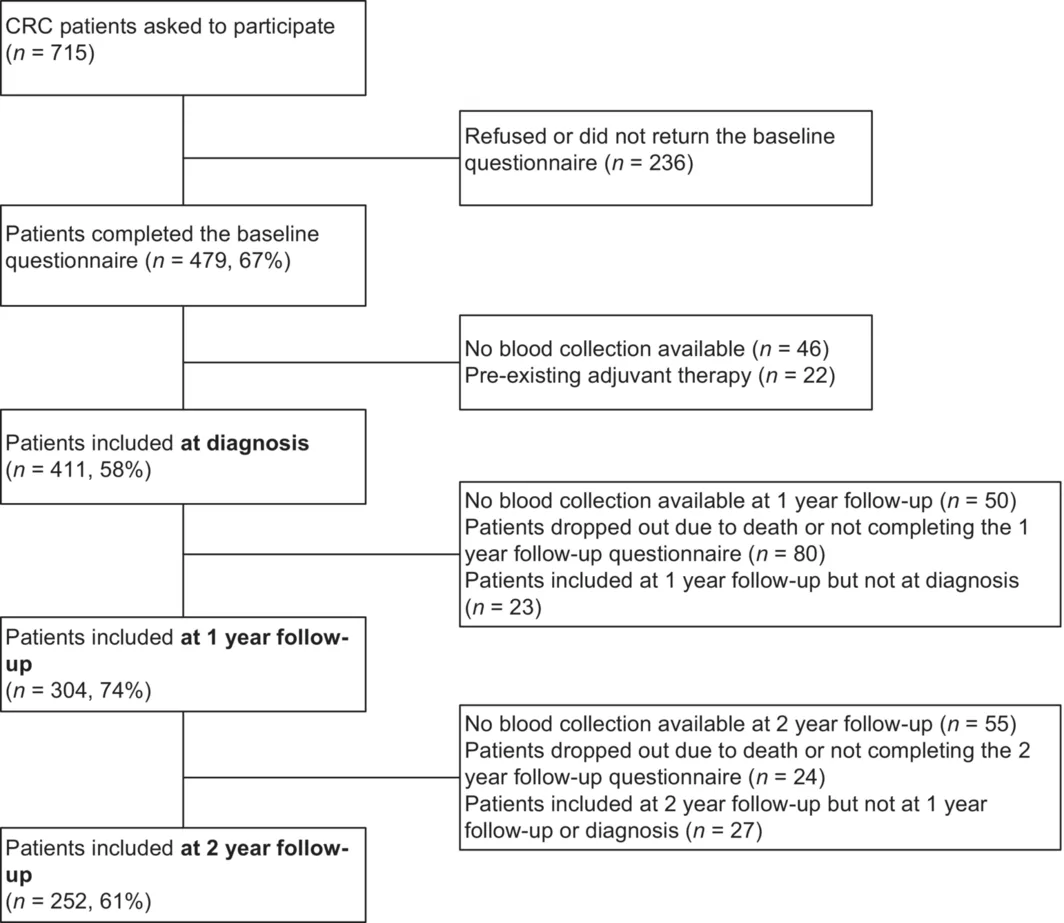
Study flow chart. Flow chart indicating the number of patients who provided data for inflammatory markers and filled in questionnaires at each assessment. Twenty‐three patients did not provide samples at baseline but did at 1‐year FU. Two patients did not provide samples at baseline and 1‐year FU but did at 2‐year FU. Two patients did not provide samples at 1‐year FU but did at 2‐year FU. This figure was previously published as fig. 1 in PMID: 41521093 DOI: 10.1016/j.clcc.2025.001.

Blood collection procedures have been described previously [15, 16]. Briefly, participants received a study package including an information letter, consent form, baseline questionnaire, and a blood draw package. The blood draw package contained tubes for blood drawing, cryovials for storing, and a short questionnaire asking about acute pre‐analytical influences that may have affected biomarker concentrations (i.e., recent caffeine/alcohol intake [24h prior to blood collection], physical activity [30 min prior to blood collection], acute illness at the time of blood collection; all yes/no questions). Both blood samples and PRO data were collected at baseline (post‐diagnosis but pre‐treatment), and at 12‐ and 24‐month follow‐ups. Figure 1 shows the sample sizes at each assessment point.

Blood was drawn via venipuncture into two blood tubes (BD Vacutainer blood tubes K2E with EDTA), one for whole blood and one for plasma, and temporarily stored (frozen at −80°C) in the hospital of administration in a uniform way following a standardized protocol. Blood samples were not taken in fasting condition. After the study had been completed (in 2022), all samples that were locally stored in the four participating hospitals were transported to the Central BioBank Maastricht UMC+. Further procedural details are provided elsewhere [16].

### 2.3 Normative Population

A cancer‐free normative sample of Dutch adults was drawn from the Health and Health Complaints projects administered in the Longitudinal Internet studies for the Social Sciences (LISS) panel (Centerdata, Tilburg University, the Netherlands) (www.centerdata.nl/liss‐panel). In short, 1027 individuals aged ≥ 18 years, matched by age and sex to a broader cancer patient population in the PROFILES registry and not specifically to the PROCORE sample, were invited. Participants received an information letter, questionnaires, and a blood draw kit following procedures identical to those used for CRC patients [16]. Participants were primarily recruited to serve as a normative reference group for two other ongoing PROFILES studies (WaTCh [12] and OPTIMUM [17]), for which ethical approval was obtained (WaTCh: NL65161.028.18; OPTIMUM: NL66913.028.18). Risk set sampling was not applied; all controls were confirmed cancer‐free at the time of selection. In total, 265 (24%) returned a complete questionnaire and a blood sample. As the original sample was not matched to the PROCORE population, stratified rematching was performed across 10 strata based on sex (two categories) and age (five groups derived by combining adjacent deciles of the age distribution of the combined sample), resulting in approximately one control per two patients (*n* = 204 controls; *n* = 411 patients). Further details on recruitment and blood collection procedures are described elsewhere [16].

### 2.4 Measures

#### 2.4.1 Sociodemographic and Clinical Factors

In the CRC sample, demographic and clinical data (i.e., age, sex, tumor stage, tumor location [colon vs. rectum], and primary treatment) were obtained from the NCR. Additional self‐reported measures included smoking status (yes/no), alcohol use (current use, recoded into yes/no based on a self‐reported questionnaire), marital status, education level, comorbidities (based on a modified Self‐Administered Comorbidity Questionnaire [18]; categorized into no, one or two or more comorbidities), and body mass index (BMI; based on self‐reported height and weight). In the normative sample, age, sex, marital status, education level, BMI, smoking status and alcohol use were self‐reported.

#### 2.4.2 Biomarkers

Eleven inflammatory biomarkers that have been involved in cancer or cancer progression [11] were measured, namely: IL‐1α, IL‐1β, IL‐6, IL‐8, IL‐10, IL‐17A, IL‐22, CRP, interferon gamma (IFN‐γ), soluble tumor necrosis factor receptor 1 (sTNFRI), and sTNFR 2 (sTNFRII).

In the CRC sample, biomarkers were measured in plasma using a custom bead‐based multiplex assay (Luminex 200TM) [19]. Normative samples were serum‐based and analyzed using immunoassay kits (MESO QuickPlex SQ 120MM system) [20]. Although alignment of serum and plasma values was explored using a convenience sample, potential bias due to coagulation‐related differences and the number of values below the detection limit precluded reliable cross‐matrix standardization. Biomarkers were therefore natural log‐transformed prior to analysis to reduce skewness, but no matrix‐based correction was applied [21]. Consequently, absolute biomarker values between CRC patients and the normative sample cannot be statistically compared.

#### 2.4.3 Fatigue

Fatigue was assessed using the MFI [22], a validated questionnaire measuring five dimensions: general, physical, mental fatigue, reduced motivation, and reduced activity. The MFI consists of 20 questions (4 items for each dimension) which are answered on a 5‐point Likert scale (1: “Yes, that is true”–5: “No, that is not true”), yielding subscale scores from 4 to 20; higher scores indicate greater fatigue. The total fatigue score can be calculated by summing all items, which range from 20 to 100.

#### 2.4.4 Confounders

Variables that were shown to have a possible influence on inflammation and/or fatigue were lifestyle variables (smoking status, alcohol consumption, BMI, and number of comorbidities) [23, 24], sociodemographic variables (age and sex) [25, 26], sleep quality [27], and pain [28], hence these factors were included in analyses for the CRC and normative sample. For the CRC sample, additionally clinical variables (days since diagnosis and cancer treatment (divided into no radiotherapy (RT) or chemotherapy (CT); RT only; CT only; RT with CT)) were taken into consideration [15].

##### 2.4.4.1 Sleep Quality

The total score of the Pittsburgh Sleep Quality Index (PSQI) was used as a measure for sleep quality. The PSQI evaluates subjective sleep quality over a 1‐month period using 19 self‐report items with 15 questions answered on a 4‐point Likert‐scale (i.e., “Not during the past month” (0) to “Three or more times a week” (3)), and 4 with open answer options (i.e., “During the past months, when have you usually gone to bed at night?”) [29]. A total score is calculated based on the 19 answered items ranging from 0 to 21, where scores > 5 are generally considered indicative of poor sleep quality [29].

##### 2.4.4.2 Pain

The pain subscale of the EORTC QLQ‐C30 [30] was used. It consists of two items measuring pain intensity and interference with daily activities over the past week. Responses are scored on a 4‐point Likert scale ranging from 1 (“Not at all”) to 4 (“Very much”). Scores were linearly transformed to a 0–100 scale, with higher scores indicating greater pain severity.

### 2.5 Statistical Analysis

#### 2.5.1 Data Preparation and Descriptive Statistics

All analyses were conducted using R (version 4.4.3). Confounders, predictors and outcome variables were compared between the CRC patients and the normative sample. Baseline sociodemographic characteristics (i.e., age, sex, and BMI) of CRC patients and the normative sample were described and compared narratively, reporting means, standard deviations, and percentages per group.

Prior to model fitting, biomarker distributions were visually inspected. As is common for inflammatory biomarkers, the distributions of all biomarkers were positively skewed (i.e., CRP, IL‐1α, IL‐1β, IL‐6, IL‐8, IL‐10, IL‐17A, IL‐22, IFN‐γ, sTNFRI, and sTNFRII), therefore a natural log‐transformation was applied. Missing biomarker values due to levels below the detection limit were imputed using multiple imputation with 1000 iterations, using a minimum of zero and a maximum corresponding to the lowest detection limit at each timepoint for each biomarker. Outliers were evaluated visually using boxplots; no values were found that exceeded the 1.5 times interquartile range. Implausible values (i.e., height of 73 cm or weight of 2 kg) were recoded as missing if the values at other timepoints did not provide any additional information. Remaining missing data were primarily due to incomplete questionnaire responses and were handled within the mixed‐model framework.

#### 2.5.2 Main Analyses Among CRC Patients

To assess how fatigue evolved over time (at diagnosis (before treatment), at 12 and 24 months follow‐ups), linear mixed‐effects models (LMMs) were fitted using the *lme4* package in R [31]. Separate models were estimated for each MFI subscale. The effect of time was modeled as an ordinal fixed effect, while a random intercept was included to model differences between patients in baseline fatigue.

To address the primary research question concerning the associations between inflammatory biomarkers and fatigue, separate LMMs were estimated for each biomarker‐MFI subscale combination. Inflammatory markers were modeled in two ways. First, overall models included the biomarker as a single time‐varying predictor. Second, hybrid models were fitted to disentangle between‐subject and within‐subject effects: for each biomarker, a person‐mean (between‐subject effect) and a person‐mean centered deviation score (within‐subject effect) were computed and simultaneously entered into the model. This allowed us to evaluate (i) whether patients with generally higher biomarker levels reported higher fatigue compared to others (between‐subject effect), and (ii) whether periods of increased inflammation within a person coexisted with periods of greater fatigue (within‐subject effect).

All models were adjusted for relevant covariates, that is, sex, age, BMI, alcohol use, smoking status, number of comorbidities, treatment, days since diagnosis at baseline, sleep quality (PSQI total score), and pain (EORTC QLQ‐C30 pain subscale). Models were estimated using the *lme4* package in R, and statistical inference for fixed effects was based on *lmerTest* [31, 32].

#### 2.5.3 Comparison With the Normative Population

To examine the association between inflammatory biomarkers and fatigue in the normative sample, separate linear regression models were fitted for each MFI subscale and each biomarker, while controlling for covariates (i.e., age, sex, BMI, comorbidities, alcohol use, smoking status, pain, and sleep quality). Linear regression analyses were performed using complete cases only (*n* = 147). Characteristics of participants with and without complete data were compared and are reported in Table S4.

Longitudinal CRC data with plasma was analyzed with LMM, whereas cross‐sectional normative data with serum was analyzed with linear regression analyses. Due to these structural differences between the two samples, associations were compared descriptively by examining the direction and magnitude of regression coefficients across groups. To aid interpretation, *z*‐tests were used to compare regression coefficients between the normative sample and each PROCORE timepoint. However, given differences in sample size and study design, these results should be interpreted with caution and are considered supplementary to the descriptive comparison.

#### 2.5.4 Multiple Testing Correction

Given the large number of tests, a false discovery rate (FDR) correction was applied separately within each fatigue‐subscale for all LMMs containing biomarkers and for the linear regression equations for the normative sample using the Benjamini–Hochberg procedure [33]. This procedure controls for the expected proportion of false‐positive results while maintaining sufficient power to detect meaningful associations. For each fatigue‐subscale, this results in an adjusted significance threshold of *p* ≤ 0.034.

## 3 Results

### 3.1 Exploratory Analyses

Demographic and clinical variables were compared between patients who did not provide a blood sample at baseline (*n* = 46) and those who did (*n* = 411). Groups were mostly comparable as *χ* ^2^‐tests (categorical variables) and independent samples *t*‐tests (continuous variables) were non‐significant (all *p*‐values > 0.068). Results can be found in the Supporting Information (Table S1). Furthermore, patients who did not provide blood samples or did not fill in the questionnaires at all timepoints (complete (*n* = 209 at baseline) vs. incomplete data (*n* = 202 at baseline)) showed slightly higher general (*p* = 0.009) fatigue and reduced motivation (*p* = 0.009) scores at baseline in comparison to patients who did provide blood samples and filled in all the questionnaires. Furthermore, patients with incomplete data showed higher mental fatigue (*p* = 0.001) at the 24‐month follow‐up in comparison to patients with complete data. Results can be found in the Table S2 and Figure S1. In the normative sample, 57 of 204 participants did not complete the PSQI and were therefore excluded from regression analyses, resulting in a complete case sample of *n* = 147. Comparison of complete and incomplete cases revealed that participants with incomplete data reported more comorbidities (no comorbidities: 10.3% vs. 24.5%; one comorbidity: 29.3% vs. 16.3%; 2 or more comorbidities: 60.3% vs. 59.2%; *p* = 0.023) and higher log‐transformed IL‐17A levels (*M* = −0.19, SD = 0.87 vs. *M* = −0.48, SD = 0.81; *p* = 0.026). All other characteristics were comparable between groups (Table S4). Lastly, initial analyses examined the correlations between acute pre‐analytical influences (i.e., caffeine intake and physical activity) and biomarker levels. Two variables (the influenza vaccination at 24‐month FU and the physical activity question at baseline) showed significant correlations with biomarker levels. However, when participants reporting these factors and high biomarker levels were temporarily removed in a sensitivity check, the correlations were no longer significant. This suggests that the observed correlations were driven by these individuals, and all participants were retained in the main analyses. These variables were therefore not included as covariates in the subsequent analyses. Results of the correlation analyses and sensitivity check can be found in the Supporting Information (Table S3).

### 3.2 Sample Characteristics

As presented in Table 1, CRC patients were on average 67 years, were predominantly not employed (*n* = 267; 65.0%), mostly married or cohabiting (*n* = 341; 83.0%), and most had two or more comorbidities (*n* = 162; 39.4%). The majority was not scheduled to receive any cancer treatment besides surgery (*n* = 263; 64%).

**TABLE 1:** Characteristics of participants.

Characteristic	Norm (*n* = 204)	CRC (*n* = 411)
Sex, no. (%)		
Male	107 (52.5)	249 (60.6)
Female	97 (47.5)	162 (39.4)
Mean age (SD), years	64.3 (11.1)	67.0 (8.9)
Education (*n* = 6 missing),^a^ no. (%)		
Low	52 (25.5)	42 (10.2)
Intermediate	66 (32.4)	263 (64.0)
High	82 (40.2)	100 (24.3)
Employment status (*n* = 19 missing), no. (%)		
Yes	84 (41.2)	125 (30.4)
No	120 (58.8)	267 (65.0)
Marital status (*n* = 1 missing), no. (%)		
Married/cohabiting	119 (58.3)	341 (83.0)
Divorced/separated	35 (17.2)	26 (6.3)
Widowed	12 (5.9)	34 (8.3)
Never married	38 (18.6)	9 (2.2)
Number of comorbidities at baseline (*n* = 4 missing), no. (%)		
0	42 (20.6)	94 (22.9)
1	41 (20.1)	122 (29.7)
≥ 2	121 (59.3)	191 (46.5)
Tumor location, no. (%)	N/A	
Colon		302 (73.5)
Rectum		109 (26.5)
Stage, no. (%)	N/A	
I		129 (31.4)
II		123 (29.9)
III		146 (35.5)
IV		12 (2.9)
Unknown		1 (0.2)
Cancer treatment, no. (%)	N/A	
No RT/CT		265 (64.0)
RT only		29 (7.1)
CT only		91 (22.1)
RT with CT		28 (6.8)
Days from diagnosis to baseline assessment,^b^ *M* (SD)	N/A	22.1 (14.1)
MFI (*n* = 2 missing), *M* (SD)		
General fatigue		
Baseline	8.7 (4.3)	8.8 (4.4)
12‐month FU		8.8 (4.2)
24‐month FU		8.7 (4.1)
Mental fatigue		
Baseline	7.2 (3.4)	8.4 (3.7)
12‐month FU		7.7 (3.7)
24‐month FU		7.9 (3.9)
Physical fatigue		
Baseline	8.5 (4.2)	8.8 (4.4)
12‐month FU		8.9 (4.3)
24‐month FU		8.7 (4.1)
Reduced activity		
Baseline	8.8 (3.7)	10.1 (4.3)
12‐month FU		9.5 (4.3)
24‐month FU		9.5 (4.1)
Reduced motivation		
Baseline	8.0 (3.4)	9.0 (3.9)
12‐month FU		8.4 (3.5)
24‐month FU		8.8 (3.7)
CRP (mg/L), *M* (SD)		
Baseline	3.4 (5.0)	1.2 (1.6)
12‐month FU		1.1 (1.6)
24‐month FU		1.5 (6.1)
IFN‐γ (pg/mL), *M* (SD)		
Baseline	8.6 (12.2)	17.7 (9.7)
12‐month FU		17.7 (9.6)
24‐month FU		18.4 (11.6)
IL‐1α (pg/mL), *M* (SD)		
Baseline	0.1 (0.6)	0.3 (2.7)
12‐month FU		0.3 (1.7)
24‐month FU		0.2 (1.6)
IL‐1β (pg/mL), *M* (SD)		
Baseline	0.1 (0.1)	3.5 (11.4)
12‐month FU		2.9 (7.4)
24‐month FU		4.0 (10.3)
IL‐6 (pg/mL), *M* (SD)		
Baseline	2.9 (17.8)	4.7 (37.0)
12‐month FU		2.5 (26.9)
24‐month FU		2.0 (12.1)
IL‐8 (pg/mL), *M* (SD)		
Baseline	15.1 (27.1)	1.8 (10.6)
12‐month FU		1.2 (8.9)
24‐month FU		1.0 (2.9)
IL‐10 (pg/mL), *M* (SD)		
Baseline	0.3 (0.2)	2.2 (2.8)
12‐month FU		2.2 (3.3)
24‐month FU		2.4 (3.1)
IL‐17A (pg/mL), *M* (SD)		
Baseline	1.0 (1.2)	3.6 (10.8)
12‐month FU		2.9 (11.0)
24‐month FU		3.0 (10.3)
IL‐22 (pg/mL), *M* (SD)		
Baseline	1.6 (7.4)	19.7 (202.6)
12‐month FU		4.2 (33.2)
24‐month FU		5.3 (41.3)
sTNFRI (pg/mL), *M* (SD)		
Baseline	3941.7 (3304.7)	1573.2 (1189.9)
12‐month FU		1619.7 (1245.5)
24‐month FU		1678.9 (1382.9)
sTNFRII (ng/mL), *M* (SD)		
Baseline	62.7 (35.9)	45.2 (33.6)
12‐month FU		45.4 (27.8)
24‐month FU		45.2 (29.7)
Mean BMI (SD), kg/m^2^		
Baseline	26.6 (4.4)	26.7 (3.9)
12‐month FU		26.1 (3.8)
24‐month FU		26.8 (3.8)
Alcohol use at baseline (*n* = 10 missing), no. (%)		
Yes	146 (71.6)	303 (73.7)
No	58 (28.5)	98 (23.8)
Smoking behavior at baseline (*n* = 9 missing), no. (%)		
Yes	16 (7.8)	47 (11.4)
No	188 (92.1)	355 (86.3)
Pain^c^ (*n* = 4 missing), *M* (SD)		
Baseline	13.8 (20.8)	9.7 (19.6)
12‐month FU		9.9 (20.2)
24‐month FU		12.1 (21.2)
Sleep quality,^d^ (*n* = 36 missing), *M* (SD)		
Baseline	3.3 (2.3)	4.9 (3.5)
12‐month FU		4.4 (3.2)
24‐month FU		4.8 (3.4)

Abbreviations: BMI, body mass index; CRP, C‐reactive protein; CT, chemotherapy; FU, follow‐up; IFN‐γ, interferon gamma; IL‐10, interleukin 10; IL‐17A, interleukin 17A; IL‐1α, interleukin‐1 alpha; IL‐1β, interleukin‐1 beta; IL‐22, interleukin 22; IL‐6, interleukin 6; IL‐8, interleukin 8; MFI, Multidimensional Fatigue Inventory; RT, radiotherapy; sTNFRI, soluble tumor necrosis factor receptor 1; sTNFRII, soluble tumor necrosis factor receptor 2.

^a^ Number of missing values is based on the CRC sample at baseline.

^b^ Patients were included at diagnosis (baseline assessment), although a few patients had their baseline assessment approximately a month after their diagnosis.

^c^ Pain is measured with the European Organisation for Research and Treatment of Cancer Quality of Life Questionnaire (EORTC‐QLQ C30) subscale “Pain.”

^d^ Sleep quality is measured with the Pittsburgh Sleep Quality Index (PSQI) total score.

Despite matching on sex and age, CRC patients were on average slightly older than the normative sample (*M* = 67.0; SD = 8.9 vs. *M* = 64.3; SD = 11.1). Furthermore, CRC patients were more often unemployed (65.0% vs. 58.8%) and more frequently married or cohabiting (83.0% vs. 58.3%) compared to the normative sample. A lower proportion of the CRC patients (46.5%) reported two or more comorbidities compared to the normative sample (59.3%).

### 3.3 Fatigue, Inflammatory Biomarkers, and Covariates

CRC survivors reported significantly higher mental fatigue across all timepoints compared to the normative sample, along with more pronounced experienced reductions in activity levels in comparison to the normative sample. Reduced motivation was also experienced more by the CRC patients than in the normative sample, especially at baseline and after 24 months, as can be seen in Table 1.

Concerning the covariates, CRC patients reported significantly poorer sleep quality across all timepoints compared to the normative population. Interestingly, CRC patients reported lower pain levels than the controls at baseline and 12‐month follow‐up, but not at the 24‐month follow‐up. Smoking prevalence was higher among CRC patients compared to the normative population, and the mean BMI of the normative sample was higher than the BMI of CRC patients at the 12‐month follow‐up, as shown in Table 1. There was no significant difference between the normative sample and CRC patients in alcohol use.

The untransformed biomarker values from the CRC patients and the normative sample can be found in Table 1. In general, the average concentrations were higher for most biomarkers among CRC patients in comparison to the normative sample at all timepoints, whereas CRP, IL‐8, sTNFRI, and sTNFRII were lower in CRC patients compared to the normative sample. These differences need to be interpreted with caution, as the biomarkers for CRC patients were measured in plasma and in serum for the normative sample and values cannot be compared one‐on‐one.

### 3.4 Trajectories of Fatigue Over Time

**FIGURE 2:**
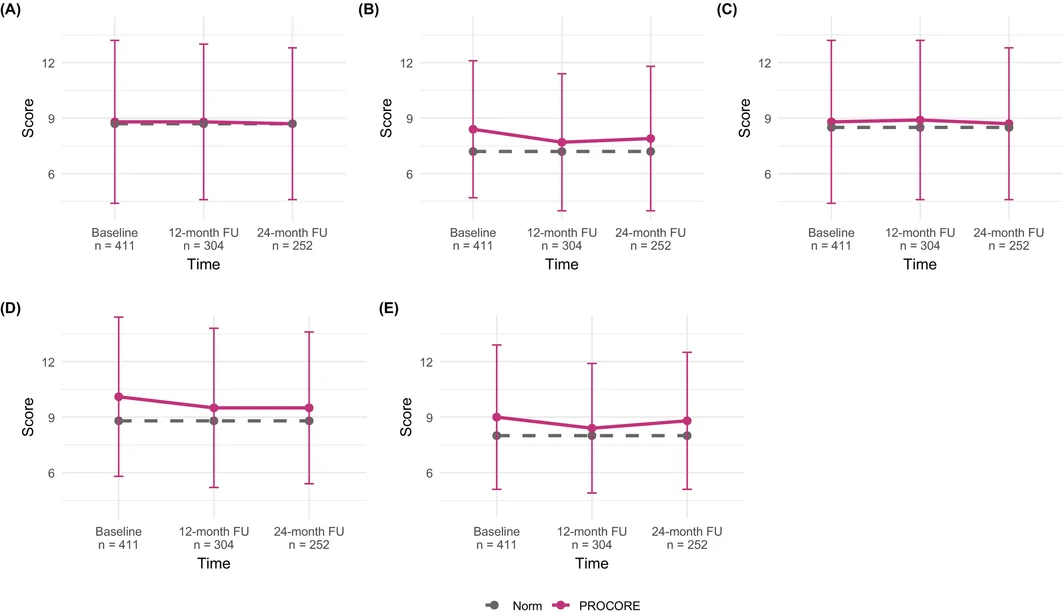
Development of fatigue over time for CRC patients and mean scores for the normative sample. (A) General fatigue. (B) Mental fatigue. (C) Physical fatigue. (D) Reduced activity. (E) Reduced motivation. Error bars represent standard deviations (CRC patients only). Numbers below the *x*‐axis indicate the number of CRC participants per timepoint. The normative sample was assessed at a single timepoint (*n* = 204).

The trajectory of CRF in CRC patients is presented in Table 2. Notably, there are no significant changes in CRF over time, except for mental fatigue, which decreased significantly from baseline to 12‐month follow‐up (*t*(600) = 3.48; *p* = 0.005). Figure 2 illustrates the CRF trajectories of CRC patients in comparison to the normative sample. CRF scores were consistently higher for the CRC patients in comparison to the normative sample for all subscales of the MFI.

**TABLE 2:** LMMs of MFI subscale scores across three timepoints for CRC patients.

Comparison	Estimate^a^	SE	df	*t*	*p* ^b^
General fatigue (MFI)					
Baseline vs. 12‐month FU	−0.22	0.22	592	−1.02	0.564
12‐month FU vs. 24‐month FU	−0.19	0.23	595	−0.80	0.701
Baseline vs. 24‐month FU	0.04	0.24	561	0.15	0.988
Mental fatigue (MFI)					
Baseline vs. 12‐month FU	0.70	0.20	600	3.48	0.002*
12‐month FU vs. 24‐month FU	0.38	0.21	605	1.77	0.182
Baseline vs. 24‐month FU	−0.32	0.22	568	−1.44	0.32
Physical fatigue (MFI)					
Baseline vs. 12‐month FU	−0.27	0.22	595	−1.22	0.441
12‐month FU vs. 24‐month FU	−0.20	0.24	599	−0.84	0.678
Baseline vs. 24‐month FU	0.07	0.25	564	0.29	0.954
Reduced activity (MFI)					
Baseline vs. 12‐month FU	0.42	0.22	596	1.91	0.138
12‐month FU vs. 24‐month FU	0.37	0.23	600	1.59	0.251
Baseline vs. 24‐month FU	−0.05	0.24	564	−0.19	0.980
Reduced motivation (MFI)					
Baseline vs. 12‐month FU	0.42	0.20	600	2.11	0.089
12‐month FU vs. 24‐month FU	0.13	0.21	604	0.59	0.826
Baseline vs. 24‐month FU	−0.30	0.22	567	−1.34	0.563

Abbreviations: df, degrees of freedom; FU, follow‐up; MFI, Multidimensional Fatigue Inventory; SE, standard error.

^a^ Estimates reflect mean differences in fatigue scores between timepoints uncorrected for relevant covariates.

^b^ All *p*‐values are derived from two‐sided Wald tests.

* Significant based on *p* value ≤ 0.034 (FDR correction).

### 3.5 Associations Between Biomarkers and Fatigue Within CRC Patients

**FIGURE 3:**
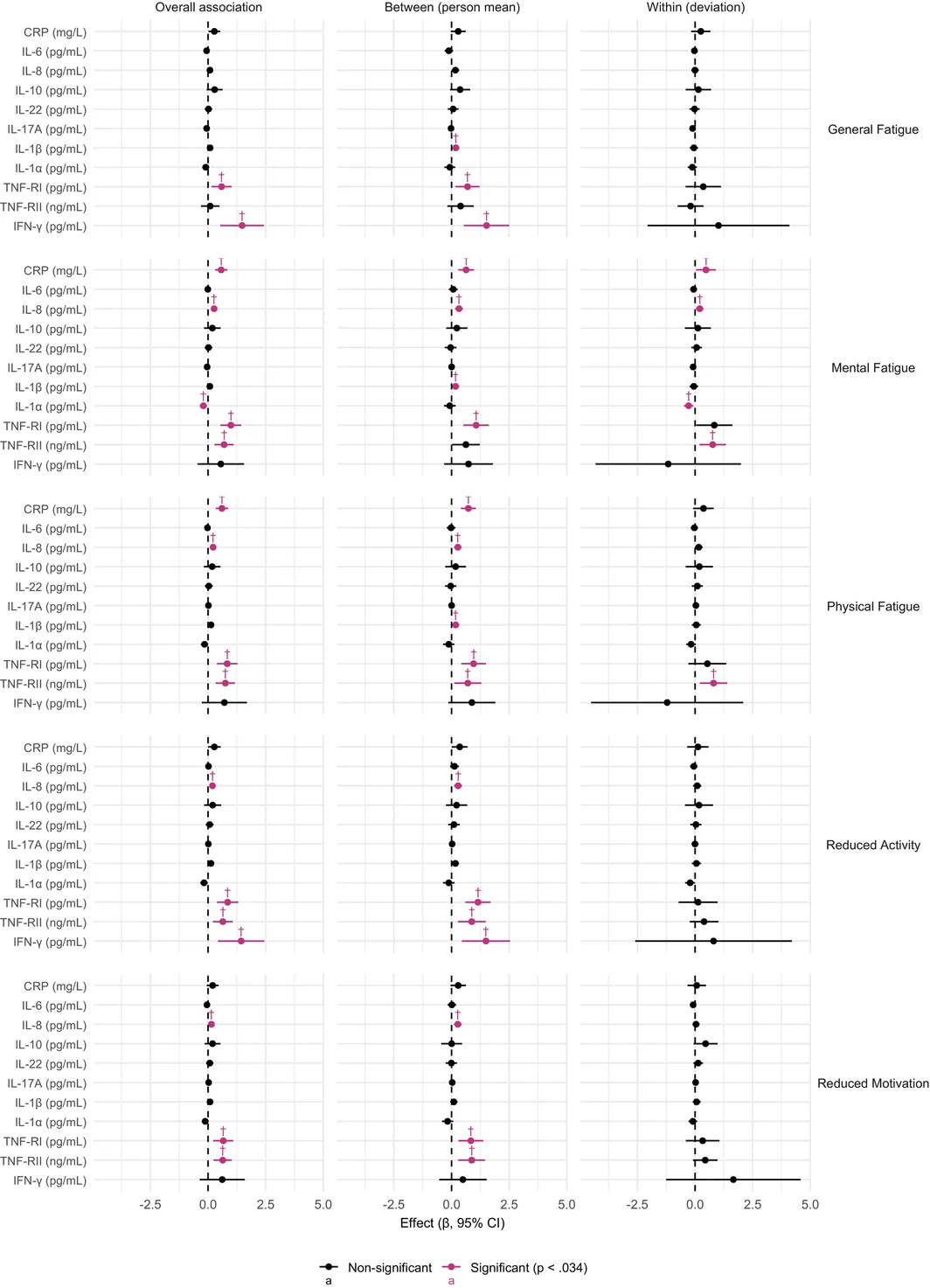
Forest plots of covariate‐adjusted hybrid LMMs between inflammatory biomarkers and MFI subscales separated by effect type. Models are adjusted for age, sex, BMI, comorbidities, days since diagnosis, treatment, pain, sleep quality, smoking, and alcohol use. All biomarkers were log‐transformed prior to analyses and separate analyses have been performed per biomarker and per MFI subscale. CI, confidence interval; CRP, C‐reactive protein; IFN‐γ, interferon gamma; IL‐10, interleukin 10; IL‐17A, interleukin 17A; IL‐1α, interleukin‐1 alpha; IL‐1β, interleukin‐1 beta; IL‐22, interleukin 22; IL‐6, interleukin 6; IL‐8, interleukin 8; sTNFRI, soluble tumor necrosis factor receptor 1; sTNFRII, soluble tumor necrosis factor receptor 2. †Significant based on *p* value ≤ 0.034 (FDR correction).

Figure 3 displays forest plots of the associations between log‐transformed inflammatory biomarkers and the five MFI subscales, separated into overall, between‐subject, and within‐subject effects. Each point represents the standardized regression coefficient (*β*) with 95% confidence interval, where positive values indicate higher fatigue scores with higher biomarker levels. Full results are provided in Table S5.

Overall, several inflammatory markers were consistently related to higher fatigue scores. In particular, sTNFRI was positively associated with all fatigue dimensions (General fatigue: *β* = 0.59 [0.15; 1.02]; Mental fatigue: *β* = 0.99 [0.54, 1.44]; Physical fatigue: *β* = 0.83 [0.38, 1.28]; Reduced activity: *β* = 0.85 [0.39, 1.31]; Reduced motivation: *β* = 0.66 [0.23, 1.09]), while CRP was positively linked to mental (*β* = 0.57 [0.31, 0.84]) and physical fatigue (*β* = 0.60 [0.34, 0.87]). Both IL‐8 and sTNFRII were associated with mental fatigue (IL‐8: *β* = 0.26 [0.14, 0.38]; and sTNFRII: *β* = 0.70 [0.28, 1.11]), physical fatigue (IL‐8: *β* = 0.22 [0.10, 0.34]; sTNFRII: *β* = 0.75 [0.33, 1.17]), reduced activity (IL‐8: *β* = 0.19 [0.07, 0.31]; sTNFRII: *β* = 0.64 [0.21, 1.07]) and reduced motivation (IL‐8: *β* = 0.15 [0.03, 0.26]; sTNFRII: *β* = 0.64 [0.24, 1.03]). IFN‐γ was significantly related to general fatigue (*β* = 1.48 [0.53, 2.42]) and reduced activity (*β* = 1.44 [0.43, 2.44]), and lower IL‐1α was associated with higher mental fatigue (*β* = −0.20 [−0.36, 0.05]).

*Between‐subjects* models, which reflect whether individuals with consistently higher inflammation across timepoints also report more fatigue, largely mirrored these findings. With additional contributions of IL‐1β to general (*β* = 0.19 [0.04, 0.34]), mental (*β* = 0.17 [0.01, 0.33]), and physical fatigue (*β* = 0.18 [0.02, 0.33]). In contrast, *within‐subjects* effects, which reflect whether a person's fatigue is higher at timepoints when their inflammation is elevated relative to their own average, were less pronounced. Only increases in CRP (*β* = 0.47 [0.05, 0.90]), IL‐8 (*β* = 0.20 [0.04, 0.37]), and sTNFRII (*β* = 0.76 [0.19, 1.33]) were significantly linked to higher mental fatigue, decreases in IL‐1α to more mental fatigue (*β* = −0.28 [−0.48, −0.08]), and increases in sTNFRII to more physical fatigue (*β* = 0.80 [0.21, 1.40]). Overall, most associations were driven by between‐subject differences, while mental fatigue uniquely reflected both between‐subject differences and within‐subject fluctuations over time.

### 3.6 Comparing CRC Patients and the Normative Population

To contextualize the findings observed in CRC patients, associations between inflammatory biomarkers and MFI subscales were also examined in the normative sample (*n* = 147; Table 3), using linear regression analyses.

**TABLE 3:** Regression coefficients from linear regression analyses for biomarker‐fatigue associations for the normative sample (*n* = 147).

MFI subscale	Inflammatory marker																	
	CRP (mg/L)^a^			IFN‐γ (pg/mL)			IL‐1α (pg/mL)			IL‐1β (pg/mL)			IL‐6 (pg/mL)			IL‐8 (pg/mL)		
	*β*	SE	CI^b^	*β*	SE	CI^b^	*β*	SE	CI^b^	*β*	SE	CI^b^	*β*	SE	CI^b^	*β*	SE	CI^b^
General fatigue	0.70	0.26	[0.18; 1.22]**	0.69	0.43	[−0.16; 1.54]	0.08	0.20	[−0.32; 0.47]	0.25	0.26	[−0.27; 0.77]	0.41	0.35	[−0.29; 1.10]	0.20	0.59	[−0.96; 1.35]
Mental fatigue	0.47	0.24	[0.00; 0.95]	−0.09	0.39	[−0.85; 0.67]	0.06	0.18	[−0.30; 0.41]	0.32	0.23	[−0.15; 0.78]	0.01	0.31	[−0.61; 0.63]	−0.004	0.52	[−1.04; 1.03]
Physical fatigue	0.55	0.26	[0.03; 0.44]	0.12	0.42	[−0.72; 0.96]	0.20	0.20	[−0.19; 0.59]	0.56	0.25	[0.06; 1.07]*	0.30	0.35	[−0.39; 0.98]	0.10	0.58	[−1.04; 1.24]
Reduced activity	0.64	0.27	[0.09; 1.18]*	0.65	0.44	[−0.22; 1.53]	0.28	0.21	[−0.13; 0.68]	0.14	0.27	[−0.40; 0.68]	0.75	0.36	[0.04; 1.46]	0.37	0.60	[−0.82; 1.56]
Reduced motivation	0.67	0.23	[0.20; 1.13]**	0.68	0.38	[−0.07; 1.43]	0.27	0.18	[−0.08; 0.62]	0.31	0.23	[−0.15; 0.77]	0.50	0.31	[−0.11; 1.12]	0.72	0.52	[−0.30; 1.74]

MFI subscale	IL‐10 (pg/mL)			IL‐17A (pg/mL)			IL‐22 (pg/mL)			sTNFRI (pg/mL)			sTNFRII (ng/mL)		
	*β*	SE	CI^b^	*β*	SE	CI^b^	*β*	SE	CI^b^	*β*	SE	CI^b^	*β*	SE	CI^b^
General fatigue	0.64	0.41	[−0.18; 1.45]	−0.17	0.38	[−0.91; 0.58]	−0.13	0.21	[−0.56; 0.29]	2.27	0.90	[0.50; 4.04]*	1.44	0.84	[−0.23; 3.11]
Mental fatigue	0.20	0.37	[−0.54; 0.93]	−0.38	0.33	[−1.04; 0.28]	0.23	0.19	[−0.15; 0.60]	1.06	0.81	[−0.55; 2.67]	0.70	0.76	[−0.80; 2.19]
Physical fatigue	−0.05	0.41	[−0.86; 0.75]	−0.48	0.37	[−1.20; 0.25]	−0.20	0.21	[−0.62; 0.21]	2.31	0.88	[0.57; 4.04]**	0.98	0.83	[−0.67; 2.62]
Reduced activity	−0.10	0.43	[−0.95; 0.74]	−0.58	0.38	[−1.34; 0.18]	−0.06	0.22	[−0.50; 0.38]	1.76	0.93	[−0.08; 3.60]	0.44	0.88	[−1.29; 2.18]
Reduced motivation	0.13	0.37	[−0.60; 0.86]	−0.61	0.33	[−1.26; 0.05]	−0.17	0.19	[−0.55; 0.21]	1.52	0.80	[−0.07; 3.11]	0.39	0.76	[−1.11; 1.88]

Abbreviations: *β*, standardized regression coefficient; CI, confidence interval; CRP, C‐reactive protein; IFN‐γ, interferon gamma; IL‐10, interleukin 10; IL‐17A, interleukin 17A; IL‐1α, interleukin‐1 alpha; IL‐1β, interleukin‐1 beta; IL‐22, interleukin 22; IL‐6, interleukin 6; IL‐8, interleukin 8; MFI, Multidimensional Fatigue Inventory; SE, standard error; sTNFRI, soluble tumor necrosis factor receptor 1; sTNFRII, soluble tumor necrosis factor receptor 2.

^a^ All biomarker concentrations are log‐transformed.

^b^ 95% confidence intervals based on linear regression analyses for all biomarker‐fatigue associations.

* Significant based on *p* value ≤ 0.034 (FDR correction).

** Significant based on *p* value ≤ 0.01.

In the normative sample, fewer and less consistent associations were observed compared to CRC patients. CRP (*β* = 0.70 [0.18; 1.22]) and sTNFRI (*β* = 2.27 [0.50; 4.04]) were associated with greater general fatigue, while IL‐1β (*β* = 0.56 [0.06; 1.07]) and sTNFRI (*β* = 2.31 [0.57; 4.04]) were related to physical fatigue. Additionally, CRP was associated with reduced activity (*β* = 0.64 [0.09; 1.18]) and reduced motivation (*β* = 0.67 [0.20; 1.13]). No significant associations were observed for mental fatigue.

When comparing patterns across groups, several distinctions emerged. Associations with mental fatigue were observed exclusively in CRC patients (i.e., CRP, IL‐8, IL‐α, sTNFRI, and sTNFRII). The normative sample showed a more limited pattern primarily involving CRP and sTNFRI. Furthermore, CRC patients exhibited broader and more consistent associations across multiple inflammatory biomarkers and fatigue dimensions, particularly for sTNFRI, sTNFRII, and IL‐8.

## 4 Discussion

This study investigated longitudinal associations between inflammatory biomarkers and multiple CRF dimensions in CRC survivors, compared with a normative sample. Inflammatory activity, particularly TNF‐α signaling (sTNFRI/sTNFRII), was consistently associated with CRF across and within individuals in the CRC sample, whereas associations were less distinct in the normative sample. This suggests that dysregulated TNF‐α pathways may play a central role in CRF [34].

Mental fatigue showed a distinctive pattern among CRC patients during the 2 years after diagnosis, remaining elevated compared to controls across all timepoints. A modest improvement during the first year may reflect recovery from the acute mental burden following diagnosis, a period typically characterized by uncertainty and emotional distress. Stabilization at 24 months likely reflects a combination of factors, including persistent low‐grade systemic inflammation, longer‐term treatment effects, and psychosocial challenges associated with reintegration into work and daily life. Multiple inflammatory markers were uniquely linked to mental fatigue in CRC survivors (higher CRP, IL‐8, sTNFRI/II; lower IL‐1α), but not in the normative sample. This suggests that cancer‐ and treatment‐related immune alterations amplify neuroimmune pathways underlying mental fatigue, highlighting a mechanism that may not be present in the general population. Inflammatory signaling can affect central nervous system functioning, contributing to cognitive difficulties such as impaired concentration and memory, which are commonly reported by cancer survivors and may further sustain mental fatigue. Sustained mental fatigue in CRC survivors is therefore likely multifactorial, reflecting the interplay of chronic inflammation and cognitive impairment [2, 8, 10, 35].

Between‐subject differences explained most biomarker‐fatigue associations among CRC patients, indicating that stable interindividual differences in inflammatory activity represent a core vulnerability to CRF. Within‐subject effects were more limited but showed that increases in CRP, IL‐8, and sTNFRII over time were accompanied by increases in mental fatigue, suggesting contributions of inflammation to persistent vulnerability and temporary symptom worsening. This suggests that individuals who chronically maintain higher inflammatory activity are more likely to experience sustained fatigue, rather than fatigue being primarily driven by transient inflammatory fluctuations within the same person. The presence of both effect types (within‐ and between‐subject) may indicate sensitivity of mental fatigue to dynamic neuroimmune processes. Inflammatory cytokines, particularly TNF‐α and IL‐8, can cross the blood–brain barrier or signal via afferent neural pathways, thereby activating microglia and altering neurotransmitter metabolism, which may manifest as mental fatigue and cognitive dysfunction [10, 36]. These neuroimmune mechanisms may differentially affect fatigue dimensions, with peripheral cytokine signaling and acute‐phase responses (i.e., CRP, IL‐8) more closely linked to physical and general fatigue, and central neuroimmune processes more relevant to mental fatigue [3, 9, 10, 36]. It is important to note that the within‐subject associations may have been underestimated due to limited repeated biomarker assessment, as not all participants contributed data at multiple timepoints, potentially reducing statistical power. Overall, the predominance of between‐subject effects suggests the existence of subgroups characterized by strong versus weak inflammation‐fatigue associations, as suggested by Vanrusselt et al. [37]. For instance, patients with persistently elevated sTNFRI/II levels may represent a biologically distinct subgroup characterized by chronic TNF‐α signaling, potentially reflecting differences in tumor biology, treatment response or comorbidity burden. Identifying such phenotypes through inflammatory profiling at diagnosis could inform targeted monitoring and early intervention strategies.

CRC survivors exhibited a broader and more complex inflammatory‐fatigue profile than the normative sample. Whereas fatigue in controls was mainly associated with CRP and sTNFRI, associations in CRC survivors extended across biomarkers and fatigue domains. Some differences between samples were not explained by covariates, suggesting cancer(treatment)‐related effects [2]. Cancer treatments such as chemotherapy and radiotherapy are known to induce systemic inflammation through tissue damage, immune activation, and disruption of the gut microbiome, potentially creating a sustained pro‐inflammatory environment that amplifies fatigue beyond what is observed in aging‐related low‐grade inflammation in the general population [15, 38]. These findings should nonetheless be interpreted with caution given methodological differences between samples (i.e., plasma vs. serum). Coagulation during serum preparation may release additional cytokines from platelets, potentially inflating cytokine concentrations relative to plasma [21]. However, soluble TNF receptors are generally considered more stable, suggesting that the sTNFRI/II associations are less likely to be purely methodological artifacts. Future studies should ideally use identical matrices for both groups.

Previous longitudinal studies in CRC populations have yielded inconsistent inflammation‐fatigue associations [8, 39, 40]. For example, the EnCoRe study found associations between hsCRP and fatigue, but not between cytokines such as IL‐6 or TNF‐α and fatigue up to 2 years post‐treatment [8]. In contrast, the present findings implicate TNF‐α signaling rather than CRP as the most consistent correlate of CRF across domains and timepoints. This discrepancy may reflect differences in biomarker panels, inclusion of pre‐treatment data in the current study, or the use of hybrid models that disentangle between‐ and within‐subject effects. The findings align with immune‐brain interaction models proposing that tumors and treatments disrupt homeostatic regulation through hypothalamic–pituitary–adrenal (HPA) axis dysregulation, and shifts in tryptophan metabolism toward the kynurenine pathway, reducing serotonergic tone and contributing to fatigue, mood disturbance, and cognitive impairment [3, 36, 41]. While our data do not establish causality, the observed patterns lend support to this framework.

Clinically, the results underscore the relevance of inflammation as a potential target for CRF management. CRF levels were highest during the first year post‐treatment and associated with inflammatory activity, suggesting a critical window for early intervention. Anti‐inflammatory strategies, whether behavioral (i.e., exercise, cognitive behavioral therapy), nutritional (i.e., anti‐inflammatory diets), or pharmacological, could reduce fatigue chronicity in vulnerable patients [3, 42]. Different fatigue subtypes may benefit from tailored approaches: general and physical fatigue may respond best to anti‐inflammatory and physical rehabilitation strategies [43, 44], while mental fatigue may require cognitive‐behavioral or neuropsychological interventions [45]. Multidimensional fatigue screening may support individualized survivorship care planning.

Strengths of this study include its longitudinal design, pre‐treatment baseline, broad biomarker panel, and normative population. Limitations include potential residual differences between serum and plasma samples, single biomarker assessment in controls, and survivor heterogeneity in treatment exposure and recovery trajectories. More vulnerable patients may have been underrepresented, potentially attenuating observed fatigue trajectories and limiting generalizability to those with the highest symptom burden. Additionally, linear regression analyses in the normative sample were based on complete cases only (*n* = 147 of 204). Participants with incomplete data reported slightly more comorbidities and higher IL‐17A levels, suggesting that the complete cases represent a somewhat healthier subset of the normative sample. This may have resulted in a slight underestimation of inflammation‐fatigue associations in the normative sample, though the impact is considered limited given that comorbidity was included as a covariate in all models. Furthermore, some samples were stored for up to 5 years at −80°C; while this is standard practice, prolonged storage may affect the stability of certain cytokines such as CRP and IL‐6 [46]. This may have introduced measurement variability, though the impact is expected to be limited given the consistent storage conditions across participants. Additionally, blood samples were not collected in fasting conditions, which may have influenced levels of certain biomarkers (i.e., IL‐6) [47]. Besides, data on the use of non‐steroidal anti‐inflammatory drugs (NSAIDs) were not available. Given that NSAIDs can substantially reduce inflammatory marker levels and may independently affect fatigue, their use represents an unmeasured confounder that may have attenuated or obscured some of the observed inflammation‐fatigue associations [48, 49]. Lastly, although pain and sleep quality were included as covariates to control for confounding, these factors may share overlapping pathways with CRF. However, given that fatigue has been shown to remain the most central symptom in network analyses even when pain and sleep are included [2], we consider this approach justified while recognizing that some degree of overcontrol cannot be excluded.

Future research should build on the current findings in several ways. First, the observed stabilization at 24 months suggests that associations between inflammation and fatigue may persist beyond the current follow‐up period of 24 months, warranting studies with longer follow‐up to capture late effects. Second, interventional designs targeting inflammation, for example through exercise [42, 43, 44], would help establish whether inflammation causally drives CRF. Third, future studies should examine sources of interindividual variation, including genetic polymorphisms in inflammatory pathways [50], and lifestyle factors known to modulate inflammatory activity [3].

In sum, CRC survivors displayed a broader inflammatory signature of fatigue than controls. These findings support inflammation as a modifiable contributor to CRF and highlight opportunities for targeted interventions to promote sustained physical and psychological well‐being. Importantly, the dominance of between‐subject effects underscores the value of identifying biologically vulnerable subgroups, while the within‐subject component of mental fatigue points to the potential benefit of dynamic and symptom‐responsive care.

## Author Contributions

**Anouk E. C. Bruijnzeels:** conceptualization, writing – original draft, methodology, visualization, writing – review and editing. **Floortje Mols:** supervision, writing – review and editing, funding acquisition. **Katrijn Van Deun:** supervision, writing – review and editing, methodology, funding acquisition. **Dounya Schoormans:** supervision, writing – review and editing, funding acquisition, conceptualization, writing – original draft.

## Funding

The present research was supported by the Netherlands Comprehensive Cancer Organisation, Utrecht, the Netherlands; the Center of Research on Psychological disorders and Somatic diseases (CoRPS), Tilburg University, the Netherlands; the Herbert‐Simon Research Institute (HSRI), Tilburg University, the Netherlands; and an Investment Grant Large (2016/04981/ZONMW‐91101002) of the Dutch Research Council (The Hague, The Netherlands). These funders had no role in the design and conduct of the study, preparation of the manuscript, and decision to submit the manuscript for publication.

## Ethics Statement

All participants have provided written informed consent. The PROCORE study received ethical approval from the Medical Research Ethics Committees United (approval number NL51119.060.14).

## Conflicts of Interest

The authors declare no conflicts of interest.

## Supporting information


**Table S1:** Sample characteristics of CRC patients with and without a blood sample at baseline.
**Table S2:** Sensitivity analyses comparing fatigue scores between CRC patients with complete (*n* = 209 at baseline) and incomplete data (blood and questionnaires; *n* = 202 at baseline).
**Table S3:** Significant correlations between pre‐analytical variables and biomarker levels, before and after sensitivity check.
**Table S4:** Sensitivity analysis of complete versus incomplete cases (normative sample).
**Table S5:** Covariate‐adjusted overall longitudinal, within‐subject, and between‐subject associations between the MFI subscales and inflammatory biomarkers.
**Figure S1:** Mean fatigue subscale scores across timepoints for CRC patients with complete and incomplete data (blood and questionnaires).

## Data Availability

The data supporting the findings of this study are available from the PROFILES registry, and can be requested for scientific purposes on www.profilesregistry.nl. All source code is publicly available on GitHub (https://github.com/anoukbr/inflammation‐CRF‐CRC‐IJC). Further information is available from the corresponding author upon reasonable request.
