# Supplementary material for: Longitudinal Associations Between Inflammation and Multi‐Dimensional Fatigue up to 2 Years After Colorectal Cancer Diagnosis
Source: Int J Cancer. 2026 Jun 19;159(10):2437–51. doi: 10.1002/ijc.70613 (PMC13595480; doi:10.1002/ijc.70613)
Supplement: Supplementary file 1 [file IJC-159-2437-s001.pdf]

Longitudinal Associations Between Inflammation and Multi-Dimensional Fatigue up to Two Years after Colorectal Cancer Diagnosis

Anouk E.C. Bruijnzeels, Floortje Mols, Katrijn Van Deun, Dounya Schoormans

Table of contents

Tables....2
Table S1....2
Table S2....3
Table S3....3
Table S4....4
Table S5....5
Figures....8
Figure S1....8

Table S1. Sample characteristics of CRC patients with and without a blood sample at baseline.

Characteristic	Did provide blood (*n* = 411)	Did not provide blood (*n* = 46)	*p* value[a]
Sex, No. (%)			.541
Male	249 (61%)	30 (65%)	
Female	162 (39%)	16 (35%)	
Mean age (SD), years	67.02 (8.94)	69.63 (10.13)	.144
Number of comorbidities, No. (%)			.716
0	94 (23%)	9 (20%)	
1	122 (30%)	12 (27%)	
≥ 2	191 (47%)	24 (53%)	
Treatment, No. (%)			.321
No RT/CT	263 (64%)	29 (63%)	
CT only	91 (22%)	10 (22%)	
RT only	29 (7%)	1 (2%)	
RT with CT	28 (7%)	6 (13%)	
Stage, No. (%)			.328
I	129 (31%)	10 (22%)	
II	123 (30%)	13 (28%)	
III	146 (36%)	20 (43%)	
IV	12 (3%)	3 (7%)	
Unknown	1 (0%)	0 (0%)	
Education, No. (%)			.219
Low	42 (10%)	4 (9%)	
Medium	263 (65%)	25 (54%)	
High	100 (25%)	17 (37%)	
Marital status, No. (%)			.095
Married/living together	341 (83%)	39 (85%)	
Separated	26 (6%)	0 (0%)	
Widowed	34 (8%)	4 (9%)	
Never married	9 (2%)	3 (7%)	
Days from diagnosis to baseline assessment, M (SD)	22.09 (14.05)	25.26 (16.89)	.068

[a] Omnibus *p* values are from χ^2-tests for categorical variables and t-tests for continuous variables.

RT = radiotherapy; CT = chemotherapy.

* Significant based on *p* value ≤ 0.05. ** Significant based on *p* value ≤ 0.01.

Table S2. Sensitivity analyses comparing fatigue scores between CRC patients with complete (n = 209 at baseline) and incomplete data (blood and questionnaires; n = 202 at baseline).

**MFI subscale**	**Time**	**Mean incomplete data**	***N* incomplete data**	**Mean complete data**	***N* complete data**	**Δ**	***t***	***p***
General fatigue	Baseline	9.4	202	8.3	209	1.1	-2.62	.009**
	12MO FU	9.4	95	8.6	209	0.8	-1.44	.151
	24MO FU	9.5	43	8.5	209	1.0	-1.30	.199
Mental fatigue	Baseline	9.3	202	8.7	209	0.6	-1.63	.103
	12MO FU	8.7	95	8.2	209	0.5	-1.06	.290
	24MO FU	10.6	43	8.4	209	2.2	-3.35	.001**
Physical fatigue	Baseline	10.3	202	9.8	209	0.5	-1.17	.243
	12MO FU	10.0	95	9.2	209	0.8	-1.44	.151
	24MO FU	10.6	43	9.3	209	1.3	-1.89	.064
Reduced activity	Baseline	8.2	202	8.6	209	-0.4	1.08	.280
	12MO FU	8.1	95	7.5	209	0.6	-1.28	.201
	24MO FU	8.4	43	7.9	209	0.5	-0.78	.439
Reduced motivation	Baseline	9.4	202	8.3	209	1.1	-2.61	.009**
	12MO FU	9.5	95	8.6	209	0.9	-1.75	.081
	24MO FU	9.6	43	8.5	209	1.1	-1.36	.179

Mean differences (incomplete – complete) were calculated using Welch's t-tests based on group means, standard deviations, and sample sizes per timepoint.

MFI = Multidimensional Fatigue Inventory; MO = month; FU = follow-up; p values are unadjusted.

* Significant based on p value ≤ 0.05. ** Significant based on p value ≤ 0.01.

Table S3. Significant correlations between pre-analytical variables and biomarker levels, before and after sensitivity check.

**Pre-analytical variable**	**Biomarker**	**Original correlation**		**Correlation after sensitivity check**	
		r	*p*	*r*	*p*
Physical activity (baseline) (n = 383)	IFN-γ	.160	.002**	.061	.230
	IL-1β	.165	.001**	.032	.535
	IL-10	.193	<.001**	.064	.213
Influenza vaccination (24-month FU) (n = 239)	IL-17A	.377	<.001**	-.013	.826
	IL-6	.132	.040*	-.014	.818
	IL-8	.257	<.001**	-.012	.839
	TNFRI	.171	.008**	-.077	.196

Sensitivity check: participants reporting the pre-analytical factor and showing high biomarker levels were temporarily removed. FU = follow-up.

* Significant based on p value ≤ 0.05. ** Significant based on p value ≤ 0.01.

Table S4. Sensitivity analysis of complete vs. incomplete cases (normative sample).

Variable	Complete (*n* = 147)	Incomplete (*n* = 58)	*p* value[a]
Mean age (SD), years	64.4 (11.2)	64.0 (10.8)	.816
Sex (female), No. (%)	69 (46.9)	29 (50.0)	.810
BMI, M (SD)	26.4 (4.5)	27.1 (4.1)	.349
Number of comorbidities, No. (%)			.023*
0	36 (24.5)	6 (10.3)	
1	24 (16.3)	17 (29.3)	
≥ 2	87 (59.2)	35 (60.3)	
Smoking, No. (%)			.075
Yes	15 (10.2)	1 (0.7)	
No	132 (89.8)	57 (98.3)	
Alcohol use, No. (%)			.963
Yes	104 (70.7)	42 (72.4)	
No	43 (29.3)	16 (27.5)	
Sleep quality[b], M (SD)	3.3 (2.3)	3.0 (N/A)	N/A
Pain[c], M (SD)	14.4 (21.4)	12.1 (18.9)	.470
MFI, M (SD)			
General Fatigue	8.4 (4.4)	9.6 (4.1)	.088
Mental Fatigue	7.0 (3.5)	8.0 (3.2)	.054
Physical Fatigue	8.5 (4.4)	8.7 (4.0)	.759
Reduced Activity	8.7 (3.8)	8.8 (3.5)	.858
Reduced Motivation	7.8 (3.3)	8.5 (3.8)	.222
CRP, M (SD)	.51 (1.2)	.58 (1.1)	.736
IFN-γ, M (SD)	1.8 (.70)	1.8 (.90)	.757
IL-1α, M (SD)	-3.6 (1.5)	-3.6 (1.5)	.776
IL-1β, M (SD)	-3.4 (1.1)	-3.6 (1.4)	.392
IL-6, M (SD)	-.19 (.95)	-.17 (1.1)	.908
IL-8, M (SD)	2.5 (.52)	2.5 (.41)	.551
IL-10, M (SD)	-1.5 (.72)	-1.5 (.54)	.745
IL-17A, M (SD)	-.48 (.81)	-.19 (.87)	.026*
IL-22, M (SD)	-.93 (1.4)	-1.0 (1.4)	.666
sTNFRI, M (SD)	8.2 (.36)	8.2 (.28)	.519
sTNFRII, M (SD)	4.1 (.39)	4.1 (.32)	.586

[a] Omnibus *p* values are from χ^2-tests for categorical variables and t-tests for continuous variables.

[b] Pain is measured with the European Organisation for Research and Treatment of Cancer Quality of Life Questionnaire (EORTC-QLQ C30) subscale 'Pain'.

[c] Sleep quality is measured with the Pittsburgh Sleep Quality Index (PSQI) total score.

MFI = Multidimensional Fatigue Inventory; CRP = C-reactive protein; IFN-γ = interferon gamma; IL-1α = interleukin-1 alpha; IL-1β = interleukin-1 beta; IL-6 = interleukin 6; IL-8 = interleukin 8; IL-10 = interleukin 10; IL-17A; interleukin 17A; IL-22 = interleukin 22; sTNFRI = soluble tumor necrosis factor receptor 1; sTNFRII = soluble tumor necrosis factor receptor 2; BMI = Body Mass Index.

* Significant based on *p* value ≤ 0.05. ** Significant based on *p* value ≤ 0.01.

Table S5. Covariate-adjusted overall longitudinal, within-subject, and between-subject associations between the MFI subscales and inflammatory biomarkers.

		Inflammatory marker						
**MFI subscale**		CRP[a] (mg/L)	IFN-γ[a] (pg/mL)	IL-1α[a] (pg/mL)	IL-1β[a] (pg/mL)	IL-6[a] (pg/mL)	IL-8[a] (pg/mL)	IL-10[a] (pg/mL)
General fatigue	Adjusted[1]	.27 [.02, .53]	**1.48 [.53, 2.42]**	-.10 [-.25, .05]	.09 [-.03, .21]	-.06 [-.18, .05]	.08 [-.03; .20]	.29 [-.06, .63]
β [95%-CI]		.035	**.002****	.193	.128	.286	.155	.104
p[b]	Within[2]	.25 [-.17, .67]	1.02 [-2.06, 4.09]	-.12 [-.31, .08]	-.05 [-.23, .13]	-.03 [-.17, .67]	.00 [-.16, .16]	.15 [-.41, .70]
		.243	.516	.237	.565	.723	.991	.604
	Between[3]	.29 [-.03, .61]	**1.52 [.54, 2.51]**	-.07 [-.31, .16]	**.19 [.04, .34]**	-.12 [-.30, .07]	.17 [.01, .33]	.37 [-.06, .81]
		.078	**.003****	.540	**.014***	.209	.043	.094
Mental fatigue	Adjusted[1]	**.57 [.31, .84]**	.55 [-.45, 1.56]	**-.20 [-.36, -.05]**	.08 [-.04, .20]	-.01 [-.13, .11]	**.26 [.14, .38]**	.19 [-.17, .55]
β [95%-CI]		**<.001****	.280	**.012***	.209	.859	**<.001****	.310
p[b]	Within[2]	**.47 [.05, .90]**	-1.16 [-4.32, 1.99]	**-.28 [-.48, -.08]**	-.05 [-.24, .13]	-.06 [-.21, .09]	**.20 [.04, .37]**	.12 [-.44, .69]
		**.030***	.469	**.006****	.573	.415	**.015***	.670
	Between[3]	**.63 [.29, .97]**	.74 [-.32, 1.79]	-.08 [-.33, .17]	**.17 [.01, .33]**	.07 [-.12, .27]	**.32 [.15, .50]**	.23 [-.23, .69]
		**<.001****	.172	.546	**.033***	.450	**<.001****	.329
Physical fatigue	Adjusted[1]	**.60 [.34, .87]**	.71 [-.27, 1.69]	-.15 [-.31, .01]	.13 [.01, .25]	-.03 [-.15, .10]	**.22 [.10, .34]**	.18 [-.18, .54]
β [95%-CI]		**<.001****	.156	.063	.036	.680	**<.001****	.327
p[b]	Within[2]	.37 [-.08, .81]	-1.21 [-4.51, 2.09]	-.17 [-.38, .04]	.06 [-.14, .25]	-.03 [-.19, .13]	.16 [-.01, .33]	.19 [-.40, .78]
		.107	.471	.105	.576	.716	.068	.535
	Between[3]	**.73 [.40, 1.06]**	.88 [-.14, 1.90]	-.12 [-.37, .12]	**.18 [.02, .33]**	-.02 [-.21, .17]	**.27 [.10, .44]**	.18 [-.27, .62]
		**<.001****	.091	.330	**.025***	.835	**.001****	.440
Reduced activity	Adjusted[1]	.27 [-.00, .55]	**1.44 [.43, 2.44]**	-.17 [-.34, -.01]	.13 [.00, .25]	.02 [-.10, .15]	**.19 [.07, .31]**	.20 [-.17, .57]
β [95%-CI]		.051	**.005****	.036	.049	.741	**.002****	.286
p[b]	Within[2]	.12 [-.34, .59]	.80 [-2.59, 4.20]	-.22 [-.43, .00]	.06 [-.14, .26]	-.05 [-.22, .11]	.09 [-.08, .27]	.17 [-.44, .78]
		.598	.642	.050	.549	.516	.302	.580
	Between[3]	.35 [.01, .70]	**1.49 [.45, 2.54]**	-.12 [-.37, .13]	.17 [.01, .33]	.13 [-.07, .32]	**.28 [.11, .46]**	.22 [-.24, .68]
		.042	**.005****	.349	.041	.199	**.001****	.352
Reduced motivation	Adjusted[1]	.20 [-.06, .46]	.62 [-.36, 1.59]	-.12 [-.27, .03]	.09 [-.03, .20]	-.05 [-.16, .06]	**.15 [.03, .26]**	.20 [-.15, .54]
β [95%-CI]		.125	.216	.103	.145	.400	**.011***	.263
p[b]	Within[2]	.07 [-.32, .47]	1.66 [-1.25, 4.58]	-.09 [-.28, .09]	.07 [-.10, .24]	-.08 [-.22, .06]	.04 [-.11, .20]	.46 [-.07, .98]
		.715	.263	.316	.433	.247	.580	.086
	Between[3]	.29 [-.05, .62]	.49 [.01, .70]	-.17 [-.42, .07]	.10 [-.05, .26]	.01 [-.17, .20]	**.27 [.10, .44]**	.00 [-.45, .45]
		.090	.042	.169	.204	.880	**.002****	.985
**MFI subscale**		IL-17A[a] (pg/mL)	IL-22[a] (pg/mL)	sTNFRI[a] (pg/mL)	sTNFRII[a] (ng/mL)			

General fatigue	Adjusted[1]	-.06 [-.15, .03]	.02 [-.14, .18]	**.59 [.15, 1.02]**	.09 [-.31, .49]
β [95%-CI]		.193	.796	**.008****	.646
p^b	Within[2]	-.11 [-.24, .03]	-.02 [-.24, .20]	.36 [-.41, 1.13]	-.19 [-.75, .37]
		.123	.847	.362	.496
	Between[3]	-.02 [-.15, .10]	.07 [-.17, .31]	**.69 [.17, 1.22]**	.39 [-.18, .96]
		.729	.560	**.010****	.178
Mental fatigue	Adjusted[1]	-.04 [-.14, .06]	.02 [-.15, .19]	**.99 [.54, 1.44]**	**.70 [.28, 1.11]**
β [95%-CI]		.430	.814	**<.001****	**.001****
p^b	Within[2]	-.09 [-.22, .05]	.07 [-.16, .29]	.83 [.05, 1.62]	**.76 [.19, 1.33]**
		.228	.551	.037	**.009****
	Between[3]	.00 [-.13, .14]	-.04 [-.29, .21]	**1.07 [.51, 1.62]**	.62 [.02, 1.23]
		.951	.754	**<.001****	.043
Physical fatigue	Adjusted[1]	.02 [-.08, .12]	.03 [-.13, .20]	**.83 [.38, 1.28]**	**.75 [.33, 1.17]**
β [95%-CI]		.716	.701	**<.001****	**<.001****
p^b	Within[2]	.04 [-.11, .18]	.10 [-.14, .33]	.53 [-.29, 1.35]	**.80 [.21, 1.40]**
		.629	.417	.201	**.008****
	Between[3]	.00 [-.13, .13]	-.04 [-.28, .21]	**.96 [.42, 1.49]**	**.70 [.12, 1.29]**
		.953	.771	**<.001****	**.019***
Reduced activity	Adjusted[1]	.01 [-.09, .11]	.07 [-.10, .24]	**.85 [.39, 1.31]**	**.64 [.21, 1.07]**
β [95%-CI]		.787	.428	**<.001****	**.004****
p^b	Within[2]	.00 [-.15, .15]	.04 [-.21, .28]	.13 [-.72, .98]	.39 [-.23, 1.01]
		.983	.764	.760	.215
	Between[3]	.03 [-.11, .16]	.10 [-.15, .36]	**1.15 [.60, 1.69]**	**.88 [.28, 1.48]**
		.702	.413	**<.001****	**.004****
Reduced motivation	Adjusted[1]	.03 [-.06, .12]	.08 [-.08, .23]	**.66 [.23, 1.09]**	**.64 [.24, 1.03]**
β [95%-CI]		.555	.349	**.003****	**.002****
p^b	Within[2]	.03 [-.10, .15]	.14 [-.07, .34]	.33 [-.40, 1.06]	.44 [-.09, .97]
		.702	.201	.376	.103
	Between[3]	.03 [-.10, .16]	-.01 [-.25, .24]	**.84 [.30, 1.38]**	**.87 [.29, 1.46]**
		.648	.946	**.002****	**.003****

Models are adjusted for age, sex, BMI, comorbidities, days since diagnosis, treatment, pain, sleep quality, smoking, and alcohol use. Time included.

[a] All biomarkers are log- and z-transformed prior to analysis.

[b] All p-values are derived from two-sided Wald-tests.

[1] The β-coefficients represent the overall longitudinal difference in inflammatory biomarker concentration using linear mixed models and can be interpreted as the amount of SD difference in log- and z-transformed biomarker concentration according to 1 SD higher score on the corresponding MFI subscale.

[2] The β-coefficients represent the change in SD units of biomarker concentration over time within individuals using a hybrid model within linear mixed models.
[3] The β-coefficients represent the change in SD units of biomarker concentration over time between individuals using a hybrid model within linear mixed models.
CRP = c-reactive protein; IFN-γ = interferon gamma; IL-1α = interleukin-1 alpha; IL-1β = interleukin-1 beta; IL-6 = interleukin 6; IL-8 = interleukin 8; IL-10 = interleukin 10; IL-17A; interleukin 17A; IL-22 = interleukin 22; sTNFRI = soluble tumor necrosis factor receptor 1; sTNFRII = soluble tumor necrosis factor receptor 2; MFI = Multidimensional Fatigue Inventory; CI = Confidence Interval.
Bold numbers indicate a statistically significant result according to the FDR-corrected *p* value ($p \leq 0.034$).
* Significant based on *p* value ≤ 0.034 (FDR correction). ** Significant based on *p* value ≤ 0.01.

Figure S1. Mean fatigue subscale scores across timepoints for CRC patients with complete and incomplete data (blood and questionnaires).

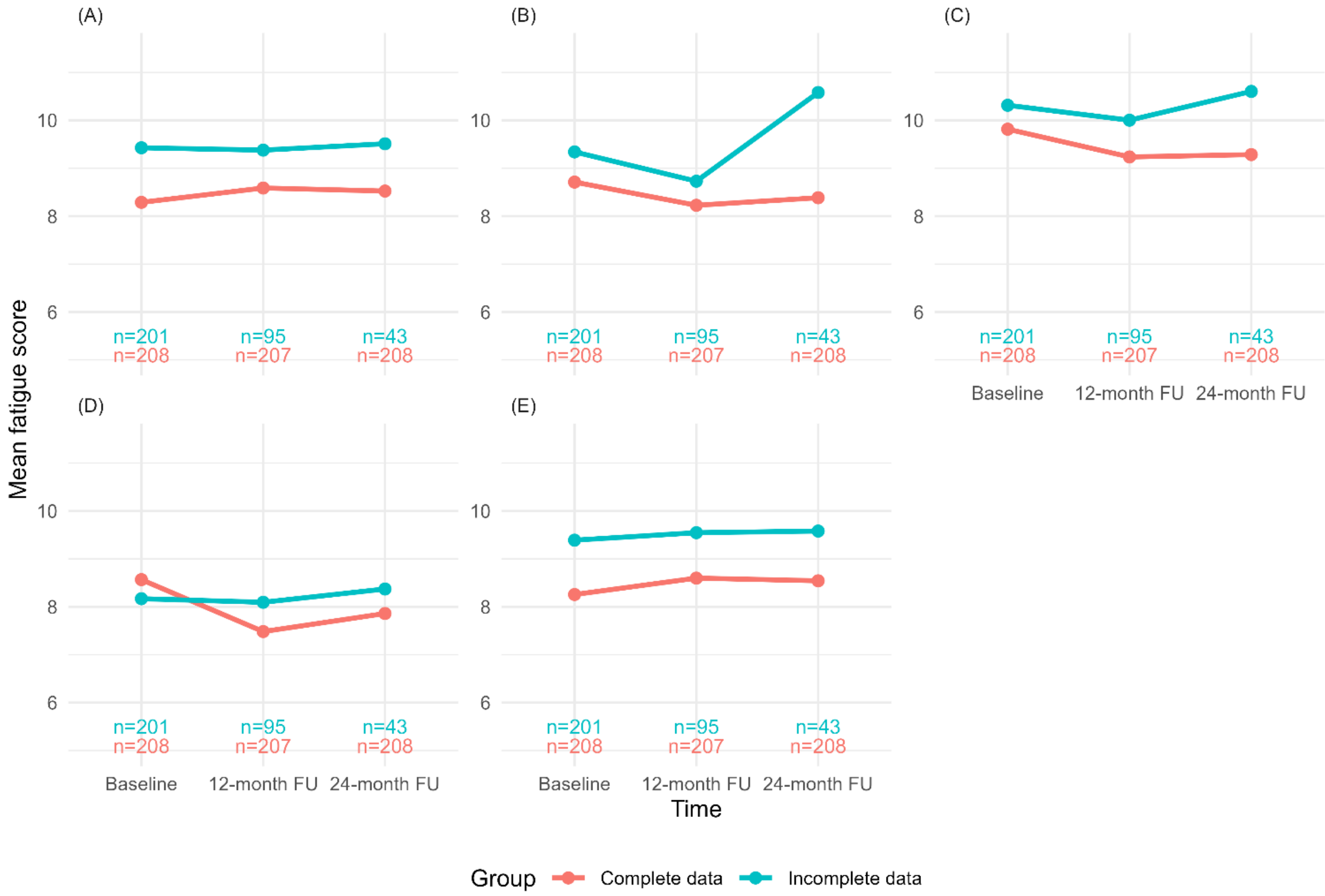

Note. (A) General fatigue. (B) Mental fatigue. (C) Physical fatigue. (D) Reduced activity. (E) Reduced motivation.
